# Acanthosis Nigricans: Pointer of Endocrine Entities

**DOI:** 10.3390/diagnostics12102519

**Published:** 2022-10-17

**Authors:** Andreea-Maria Radu, Mara Carsote, Mihai Cristian Dumitrascu, Florica Sandru

**Affiliations:** 1 Department of Dermatovenerology, C. Davila University of Medicine and Pharmacy & ”Elias” University Emergency Hospital, 011863 Bucharest, Romania; 2 Department of Endocrinology, C. Davila University of Medicine and Pharmacy & C.I. Parhon National Institute of Endocrinology, 011863 Bucharest, Romania; 3 Department of Obstetrics and Gynaecology, C. Davila University of Medicine and Pharmacy & University Emergency Hospital, 011863 Bucharest, Romania

**Keywords:** acanthosis nigricans, insulin resistance, obesity, diabetes mellitus, polycystic ovary syndrome, metabolic syndrome, glucose, endocrine

## Abstract

Acanthosis nigricans (AN) has been reported in relation to insulin resistance (IR). We aim to review AN through an endocrine and metabolic perspective focusing on IR in association with metabolic complications such as obesity, diabetes mellitus (DM), and metabolic syndrome (MS) with/without polycystic ovary syndrome (PCOS). We revised English papers on PubMed covering publications from the last 5 years. The current prevalence of AN varies from 4.5 to 74% (or even 100%, depending on the studied population), with equal distribution among females and males. Despite higher incidence with an age-dependent pattern, an alarming escalation of cases has been noted for obesity and MS in younger populations. Most frequent IR-associated sites are the neck, axilla, and knuckles, but unusual locations such as the face have also been reported. Quantitative scales such as Burke have been used to describe the severity of the dermatosis, particularly in correlation with IR elements. Dermoscopic examination are required, for instance, in cases with sulcus cutis, hyperpigmented spots, crista cutis, and papillary projections. A skin biopsy may be necessary, but it is not the rule. Both IR that clinically manifests with or without obesity/MS correlates with AN; most studies are cross-sectional, with only a few longitudinal. The approach varied from screening during school periodic checkups/protocols/programs to subgroups of individuals who were already known to be at high cardio-metabolic risk. AN was associated with type2DM, as well as type 1DM. Females with PCOS may already display metabolic complications in 60–80% of cases, with AN belonging to the associated skin spectrum. AN management depends on underlying conditions, and specific dermatological therapy is not generally required, unless the patient achieves metabolic control, has severe skin lesions, or desires cosmetic improvement. In IR cases, lifestyle interventions can help, including weight control up to bariatric surgery. In addition, metformin is a key player in the field of oral medication against DM type 2, a drug whose indication is extended to PCOS and even to AN itself, outside the specific panel of glucose anomalies. In terms of cosmetic intervention, limited data have been published on melatonin, urea cream, topical retinoids, vitamin D analogs, or alexandrite laser. In conclusion, awareness of IR and its associated clinical features is essential to provide prompt recognition of underlying conditions. AN represents a useful non-invasive surrogate marker of this spectrum in both children and adults. The pivotal role of this dermatosis could massively improve endocrine and metabolic assessments.

## 1. Introduction

Currently, insulin resistance (IR) represents an endocrine/metabolic issue with a high prevalence in the general population, frequently associated with conditions such as obesity, diabetes mellitus (DM), dyslipidemia-like hypertriglyceridemia, metabolic syndrome (MS), hepatic steatosis, polycystic ovary syndrome (PCOS), or, rarely, some endocrine tumors [1,2,3,4,5,6,7,8,9,10]. Rare IR-related entities include different types of congenital or acquired lipodystrophy syndromes (Berardinelli–Seip syndrome), mutations of insulin receptors (type A IR syndrome, leprechaunism or Donohue syndrome, and Rabson–Mendenhall syndrome), or other genetic disorders such as Alstrom syndrome, SOFT syndrome, MARFAN syndrome, and monogenetic obesity with underlying melanocortin-4 receptor (*MC4R*) gene mutations [11,12,13,14,15,16,17,18].

There are no particular studies addressing the global prevalence of IR, neither has it been represented as a diagnostic by itself, whereas the practical aspects of clinical presentation, assessments, and specific management show a heterogeneous picture depending on the manifestations of comorbid disorders and syndromes [1,2,3,4,5,6,7,8,9,10].

Awareness of IR-associated clinical features is essential to provide prompt recognition of underlying conditions and associated cardio-metabolic and reproductive issues. Prompt recognition allows access to early active interventions, mostly from an endocrine and metabolic perspective, but could also be oncological [1,2,3,4,5,6,7,8,9,10].

Among the clinical hallmarks of IR, we mention acanthosis nigricans (AN), a dermatosis that was first described in association with obesity in 1889 by Gerson Unna P. and Pollitzer S. [19,20,21,22]. Acanthosis is the thickening of the epidermis, and should be differentiated from pseudoachanthosis. The term may also be used as glycogenic acanthosis, which is a benign condition typically at the esophagus level that underlies multiple plaques of hyperplastic squamous epithelium in association with glycogen deposits [23,24,25,26,27].

AN has hyperpigmentation of a brown, dark blue, or black color, with a velvet-like texture, and typically involves areas such as the neck, axilla, and knuckles (top three most frequent sites), as well as the groin, umbilical, and perianal regions. AN has also been found in the inframammary area, as well as the antecubital and popliteal fossae, with a symmetrical pattern of distribution [28,29,30,31,32,33,34,35,36,37,38]. The same patient may display different patterns at distinct locations, or due to skin changes, in a time-dependent manner. 

Many pathogenic contributors are described in relation to signature IR, at the skin level. Insulin-like growth factor (IGF), fibroblast growth factor receptor (FGFR), and epidermal growth factor receptor (EGFR) have been recognized as promoters of epidermal keratinocyte and dermal fibroblast proliferation. These are associated with a general pro-inflammatory status that may be a contributor to AN development as well as IR-activated pathways of glucose and lipid metabolism anomalies [28,29]. IGF1 has been reported as a promotor in other endocrine disorders underlying IR, such as acromegaly. 

The currently increasing prevalence of AN varies from 4.5 to 74%, and has even been reported as 100%, depending on the studied population( e.g., diabetic or obese cohorts), with equal distribution among females and males. Despite a higher incidence with increases in age, there have been connections made with escalating cases of obesity and MS in younger populations [28,29,30,31].

AN has been reported in association with IR and other disorders, such as obesity, DM, MS, PCOS, some autoimmune diseases, malignancies (most frequently, gastrointestinal neoplasms and neuroendocrine neoplasia), endocrine tumors, such as acromegaly and Cushing’s syndrome, iatrogenic circumstances or so called drug-induced AN (for instance, local AN due to insulin injections, topic corticotherapy, etc.), and atypical presentations on the nasal crease due to local factors (as seen in patients with persistent itching caused by chronic allergic rhinitis). Thus, recognizing AN is of utmost importance [32,33,34,39,40,41].

Burke’s AN quantitative scale (launched in 1999) may be used to describe the severity of AN, particularly in correlation with IR elements; however, there are still inconsistencies among published data [42]. A new scoring system for AN severity (SCANS) was proposed in 2020, in a pilot study that assessed the texture, sites, number of lesions, etc. (a severity score between 0 and 46) of the disease [43]. On the other hand, texture grading of AN may correlate with the severity of metabolic complications, and simple identification of AN may serve as a non-invasive tool for IR screening, especially in populations who are assessed or followed for different purposes such as pediatric cohorts during scholar evaluation programs [28,29,30,31,32,33,34,35,36,37].

By having a typical (clinical) appearance, diagnosis of AN is straightforward; however, a dermoscopic examination may be required in some cases, such as cases with sulcus cutis, hyperpigmented spots, crista cutis, or papillary projections. A skin biopsy may be necessary to provide a pathological report that typically identifies epidermal hyperkeratosis, papillomatosis, and acanthosis elements [29,35,38]. Histological examination helps with differential diagnosis for similar lesions such as confluent and reticulated papillomatosis, as well as in obese children and teenagers and women with PCOS with associated AN [44,45]. Overall, most dermoscopic findings correlate with pathological reports, allowing a useful magnification to differentiate AN from other hyperpigmentation; thus, skin biopsies are not typically required [35,46,47].

When it comes to specific endocrine and metabolic evaluations, the homeostasis model assessment of insulin resistance (HOMA-IR), fasting insulin level, glucose/insulin ratio, and quantitative insulin sensitivity check index (QUICKI) may be used to confirm the presence of IR. Later on, the evaluation of MS components and associated cardiovascular, respiratory, gynecological, or even oncologic complications is necessary, using a point–by–point, multidisciplinary approach [29,36].

In terms of AN management, the treatment of the underlying disease represents a stepping stone in improving AN: nutritional and psychological intervention for weight control; bariatric surgery; insulin sensitizers for IR, usually metformin; oral contraceptives in PCOS; or gonadotropin releasing hormone agonist therapy in young populations for hyperandrogenemia [48,49,50]. There are also dermatological options (which are not typically used in every day practice), such as topical retinoids or vitamin D analogues, chemical peels (trichloroacetic acid or salicylic–mandelic acid peeling), alexandrite laser, and systemic retinoids [28,29,37,51].

We aim to overview AN using an endocrine and metabolic approach focusing on IR in association with metabolic complications such obesity, DM, and MS, with/without PCOS. 

## 2. Methods

We revised English papers on PubMed covering 5 years of the most recent publications (from 2017 to August 2022), using different combinations of key words, such as: “acanthosis” and “insulin resistance” or “diabetes mellitus”, “obesity”, “metabolic syndrome”, “polycystic ovary syndrome”, “endocrine”, “acromegaly”, “Cushing”, “cortisol”, “thyroid”, “neuroendocrine”, “neoplasia”, etc. 

Exclusion criteria were case reports or case series; specific syndromes concerning lipodystrophy (genetic or acquired); genetic IR syndromes that may manifest with AN; extreme subtypes of PCOS such as HAIR-AN syndrome; and secondary or gestational DM.

We followed three main sections concerning the clinical perspective of AN: studies on MS and its components, especially obesity; studies addressing the diabetic population; and studies on PCOS. 

## 3. AN and IR

Both IR that clinically manifests with or without obesity/MS correlates with AN, with most studies being cross-sectional, and only a few longitudinal. The correlation depends on the studied population (noting that both pediatric and adult data are provided), as well as the general approach to the patients, from screening during school, periodic checkups/protocols/programs, or professional assessments of particular subgroups who are already known to be at high cardio-metabolic risk. For instance, one study from 2022 on 1525 children aged between 9 and 12 years (Partners for Heart Health program from 2010–2018) assessed 13 cardiovascular risk factors, including AN. They found that AN was significantly higher in the group with highest levels of metabolic features [52].

Another large pediatric cohort (N = 4023; aged between 2 and 8 years) provided WC cutoffs to predict AN as a marker for IR (Children’s Healthy Living Program’s 2012–2013 in US-affiliated Pacific region) [53]. The CARDIAC project also included the examination of 52,545 fifth grade students in West Virginia to detect AN (between 2007 and 2016); AN was identified in 4.5% of cases. Among those with complete blood assays, 79% of the subjects who displayed AN had IR, whereas IR correlated with a high triglyceride/HDL-cholesterol radio and triglycerides/LDL-cholesterol ratio (*p* < 0.0001) [54]. A multi-centric population-based cross-sectional study from Brazil searched MS-associated elements among 1125 primary health care nurses; the prevalence of MS was 24.4%. The most frequent element of MS was low HDL-cholesterol, and multivariate analysis showed a statistical significant association between MS and AN (PR = 3.23, 95% CI between 2.65 and 3.92) [55].

Most frequent locations for IR are in the neck and axilla for adults, and knuckles for younger subjects. A hospital-based transversal study from 2022 on 60 patients with neck AN showed that the most frequent sites (aside from neck involvement) were the axilla (85% of individuals) and knuckles (55%); MS was positive in 78% of cases and IR criteria based on HOMA-IR was confirmed in 56.66% of the entire cohort. Although axillary AN severity (Burke’s scale) was correlated with MS (*p* = 0.001) and IR (*p* = 0.03), neck AN did not correlate with metabolic features, except for neck texture grading and IR (*p* = 0.005) [56].

A study of 148 obese children (aged between 6 and 18 years; 56.1% females) identified 39.9% cases with AN; the most frequent sites were the axilla 27% and neck 16.9%. Those with more than one site involved in AN was 55.9%. There was a correlation between AN and MS (*p* = 0.003); and among the entire cohort, 27.7% had both MS and AN [57]. According to González-Saldivar G. et al., AN in the knuckles was shown to be a trustworthy IR sign among adolescents and young adults. The study included two groups, one of 149 patients with AN in the knuckles and the other one of 145 controls, and the results showed statistically significant higher levels of fasting insulin (13.45 ± 7.8 versus 8.59 ± 3.63 µU/mL, *p* < 0.001) and HOMA-IR (2.86 ± 1.68 versus 1.78 ± 0.77, *p* < 0.001) among the first group [58].

AN scales did not correlate with each of the derivate parameters of IR; thus there are still controversies around the practical use of severity scores. One study on 336 adults patients with AN showed that AN correlated with an increased body mass index (BMI: r = 0.299, *p* < 0.001), and not with DM (*p* = 0.43), when compared with 243 heathy controls (AN free). The scoring system that we mentioned before showed a correlation with WC (waist circumference) among non-DM individuals (r = 0.131, *p* = 0.24), with respect to total cholesterol (r = 0.155, *p* = 0.04) [43]. Another study used the short and extended versions of Burke’s scale on 139 teenagers with a BMI equal to or higher than the 85th percentile, aged between 12 and 18 years old (N1 = 67 with AN versus N2 = 72 without AN). Persons from the N1 group had higher insulin and HOMA-IR levels (*p* = 0.003) compared with N2, but neither the short nor extended Burke’s scale were correlated with the degree of hyperinsulinemia and IR; however, AN as a single input predicted hyperinsulinemia in 7.3% of cases, and respective IR in 7.1% of the patients [59].

Another study on 95 obese school-aged patients showed that severity of neck AN, according to Burke’s scale, correlated with diastolic blood pressure (*p* = 0.001) and blood levels of triglycerides (*p* = 0.02), and inversely correlated with adiponectin (*p* = 0.02) [60]. The majority of data show that subgroups of subjects with AN have the most severe IR profiles, as expected. A cross-sectional study conducted in North India by Singh KS. et al. reported IR in 41.4% patients with AN (N = 70), whereas among the controls (N = 70, AN free), IR was seen in only 17.1% patients (*p* < 0.05). Moreover, AN patients had higher HOMA-IR values compared with controls (*p* < 0.05) [61]. A study on Mexican obese children aged between 2 and 16 years identified 49 infants with AN who had higher levels of serum insulin (3.67  ±  2.56 versus 2.42  ±  1.45 µU/mL, *p* =  0.005), fasting glucose (84.2  ±  12.6 versus 77 ± 9.9 mg/dL, *p* ≤ 0.001), as well as HOMA-IR (0.77  ±  0.54 versus 0.46  ±  0.28, *p* ≤ 0.001) when compared with healthy controls [62].

A different paper with a similar population (N = 628 Mexican children who were referred for obesity) showed that 79.3% had IR, and 55.4% met MS criteria, whereas AN was identified in 94.8% of individuals with IR [63]. A transversal study on 161 overweight children and teenagers included 51.5% with AN; the AN group (with similar age versus the non-AN group) had a higher BMI (*p* < 0.0001), systolic and diastolic blood pressure (*p* = 0.006, respective *p* = 0.002), and higher HOMA-IR (*p* < 0.0001), as well as lower HDL-cholesterol (*p* = 0.003) [64].

Most studies that analyzed IR–AN correlations only had a transversal component. One longitudinal Israeli study confirmed the predictive value of AN. This was a retrospective study in 230 obese children (with BMIs above the 95th percentile), aged between 6 and 17.6 years and followed for at least 1 year. Among the patients, 20.9% met the criteria of metabolically healthy obesity, which correlated with a higher IR, as defined by the presence of both AN and higher HOMA-IR. Also, AN at baseline predicted metabolically healthy obesity (OR = 2.35, *p* = 0.35) [65]. Obese insulin-sensitive individuals had more favorable cardio-metabolic profiles compared with obese insulin-resistant persons [66]. On the other hand, high numbers of glucose and insulin anomalies were found in apparently asymptomatic obese children and adults, suggesting the importance of indicators such as AN [67].

Another study following IR elements in women with premature adrenarche, a status that is prone to obesity, hyperinsulinemia, IR, dyslipidemia, and PCOS. Thirty women with PA were compared with 41 healthy controls (longitudinal study, from pre-pubescent to young adults). No differences were identified between groups concerning MS prevalence and elements such as hyperlipemia, high blood pressure, liver steatosis, fasting glycaemia, and insulin. There were significant differences found, however, for IR (*p* = 0.014) and AN (*p* = 0.01) [68].

Another longitudinal study included 34 girls with premature adrenarche who were followed until they reached their final height (from the age of 15.2 years to 28.2 years). The cohort presented with obesity (11.8%), high blood pressure (8.8%), hyperinsulinemia (29.4%), abnormal HOMA-IR (38.2%), and AN (14.7%) [69]. Pro-inflammatory cytokines and adipokines have been extensively studied in the obese population, including in patients with MS; however, the panel is very complex and direct practical applications in daily clinical activity are limited [70].

AN was analyzed in relation to chemerin, a pro-inflammatory adipokine that is a contributor to MS anomalies, in a case-control study on 25 adults with obesity and AN, 25 patients with obesity (AN free), and 25 healthy controls. As seen in some studies, the neck was the most frequent site (80%) of AN, followed by the axilla (68%); chemerin was statistically significant higher in patients with obesity versus controls, regardless of AN. Of the patients with AN, 100% met MS criteria, suggesting that AN may serve as a non-invasive marker of MS. This would make AN an easy-to-apply clinical measure that may prove useful in addition to different blood markers of IR or associated inflammation [71].

Exploring AN in association with anthropometric parameters and Tanner stages, as part of clinical evaluations, as well as glucose and lipid blood profiles (as blood assays) in the pediatric population represent the most useful tools for IR assessment. AN was studied in 670 Mexican American children (aged between 6 and 17 years, 49% girls) as a composite marker of cardiometabolic risk; 33% of this non-DM cohort had AN. AN was used as a quasi-quantitative parameter based on a severity score ranging from 0 to 5. The results concerning AN included: a heritability of 0.75 (*p* < 0.0001); positive correlations with BMI, HOMA-IR, and high-sensitivity C reactive protein (*p* < 0.05); and negative correlations with HDL-cholesterol and physical fitness score [72]. Another study that included 119 children with MS and 426 children without MS found a higher prevalence of AN in the first group (89.9 versus 34.03%), according to higher HOMA-IR levels (5.47 ± 0.17 versus 2.18 ± 0.04, *p* < 0.05) [73].

Another study (N = 60 subjects with AN, aged between 2 and 24 years) also confirmed that overweight and obese patients had higher HOMA-IR2, but the association between AN and IR was found in normal weighted individuals as well (*p* = 0.045) [74]. Currently, we do not have enough data concerning the IR prediction power of AN in normal-weighted prepubescent individuals, as opposed to obese persons of the same age. Hence, this remains an open issue.

In contrast to the most frequent sites of AN (neck, axilla, and knuckles), facial AN (FAN) does not have the same clinical presentation, often being misdiagnosed, as it is not in typical AN locations and can be confused with other pigmented lesions of the face. AN should be suspected in cases with obesity and de novo facial pigmentations. An association between FAN and IR has also been suggested. Verma S. et al. previously reported a high prevalence of IR (82.1%) and obesity (87.5% in males and 100% in women) among FAN patients [75]. Recently, Shah VH. et al. showed that FAN displayed different patterns such as a hyperpigmented band over the forehead (55%); periorbital, respective perioral darkening (25%, respective 10%); or even generalized darkening (10%). This was a study on 40 patients with FAN (men/women ratio of 24/16) and 40 healthy normal-weighted controls. All FANs were investigated through dermoscopy, which identified crista cutis of linear patterns or hyperpigmented dots and sulcus cutis. Biopsy results (20/40 patients) showed hyperkeratosis, papillomatosis, acanthosis, and high basal melanin. Individuals with FAN had statistically significant higher HOMA-IR, insulin levels, fasting glycaemia, triglycerides, and total cholesterol (*p* < 0.05) compared with the control group [76]. Nevertheless, as single facial lesions or multi-sited AN, FAN represents a subtle index for the same IR-related metabolic picture as other AN locations. FAN should be differentiated from other causes of melanosis—for instance, periorbital melanosis is also reported in individuals with IR—and hyperinsulinemia-associated complications [77]. A study on 300 Indian adult men with facial hyperpigmentation (aged between 18 and 74 years, average age of 37.35 years) showed the following entities: melasma (76.7%)—which is mostly sun-induced; periorbital hypermelanosis (10.7%); lentigens and freckles (together representing 8.7%); AN (4%), lichen planus (3.3%), and respective contact dermatitis (3%). AN was correlated with obesity and DM [78].

Another dermatologic element that has been reported to be associated with IR is lichen planus [79]. However, there was only one study focusing on this lesion in association with AN and acrochordon: a cohort study of 108 patients with lichen planus was compared with 109 controls. The prevalence of MS was higher in the first group (50.9 versus 36.7%, *p* = 0.3), AN incidence was higher in the lichen group (*p* = 0.009), and MS was significantly more frequent in patients with AN + lichen, suggesting that AN is predictive of MS in patients who are already identified with lichen planus [80].

Of note, AN was studied in 40 obese children, in addition to anthropometric parameters, metabolic features, and a questionnaire-based assessment of lower urinary tract dysfunction that was confirmed in 19% of cases. Although metabolic lab tests were not correlated with the presence of urinary symptoms, AN was, pointing to a 1.75-fold (*p* < 0.05) increased risk of urinary dysfunction [81]. Further studies are needed to confirm the use of AN as a predictor for urinary dysfunction in obese pediatric populations.

In terms on interventional trials for patients diagnosed with AN, a double-blind, randomized (1:1), active-controlled study of 33 patients with AN administered either 500 mg metformin or a combination of alpha-lipoic acid with biotin, calcium, and zinc sulfate (metformin and alpha-lipoic acid are insulin-sensitizing drugs) for 12 weeks. Both groups showed effective improvements in severity and texture of neck AN. This was in association with a statistically significant reduction in BMI, fasting glucose, and lipid profile [82]. Also, a interventional type (open design) pilot study with a small sample size showed that 17 patients with AN showed significant improvements in AN score in the neck and axilla following 12 weeks of daily melatonin (3 mg), as well as improved BMI, body fat, inflammatory markers, HOMA-IR, and fasting insulin [83]. However, further studies are necessary to confirm the practical importance of melatonin therapy regarding IR. 

A study performed on 40 teenagers with neck AN showed an improvement after 8 weeks of a topic application of 20% urea cream, which was well-tolerated and more efficient than a lower concentration of 10% [84]. Another type of intervention directly concerning obesity was bariatric surgery. A study of 65 men with obesity offered laparoscopic sleeve gastrectomy and showed that patients with obesity without AN (N = 20) had less severe metabolic parameters at baseline compared with patients with obesity and AN (N = 45); within one year since surgery, AN score was significantly reduced, and was correlated with significant improvements in BMI, serum insulin, HOMA-IR, inflammatory status, and total testosterone [85]. Moreover, it was suggested that weight control programs in children should be started before they developed AN [86]. (Table 1).

## 4. AN and DM

AN has been studied in relationship with DM type 2, as part of a larger spectrum underlying IR-MS, and also with DM type 1 [87,88,89,90]. One study from 2022 on 347 diabetic persons (DM type 1 and 2) identified a prevalence of 71% regarding dermatological lesions aside from infections, pregnancy, cancer, iatrogenic effects, renal failure, and incidental rheumatologic and other endocrine conditions. AN was found among the most frequent skin involvements, as well as pruritus, acrochordon, and diabetic dermopathy, regardless of the type of DM; these dermatological lesions were correlated with female sex, DM duration, presence of obesity, and poor glycemic control [91]. MODY (maturity-onset diabetes of the young) is often misdiagnosed as DM type 1 or 2. One registry-based study from 2022 revealed an AN prevalence of 12.5% at DM diagnosis [92]. Another cohort of 100 subjects with DM type 2 diagnosed before the age of 40 years (mean age at DM diagnostic of 32.5 ± 5.5 years) showed an AN prevalence of 12% (after an average DM duration of 7.7 ± 3.8 years) [93]. Higher AN presence was reported by a study from Ghana on 106 diabetic patients (children and teenagers): 15% had DM type 2 and all of them displayed AN [94]. 

Similarly, one study on 104 children aged less than 15 years (New Zealand) diagnosed with DM type 2 showed a 90% prevalence of AN (at mean age of 12.9 years) [95]. A positive family history for DM was confirmed to increase the risk of developing AN in obese children. This was an analytical study on 400 teenagers, aged between 13 and 14 years (mean age of 13.31 ± 0.46 years), who had an AN prevalence of 14.5%, which was higher in those with family history of either type of DM (21.18%), whereas the highest AN ratio was of 61.54% in obese individuals [96]. In 2019, Azizian Z. et al. ran a 225-patient cross-sectional study that showed that cutaneous lesions, including AN, were strongly linked to patient age and DM duration (*p* < 0.05) [97]. 

Remarkably, a diabetic patient under insulin therapy may develop AN at the injection site, as well as lipodystrophy or amyloidosis [98,99,100]. Calcaterra V. et al. carried out a retrospective study on 138 patients newly diagnosed with DM type 1. Comparing the patients with AN (N = 7) with the 131 AN-free persons, the AN positive patients had higher BMIs (23.86 ± 4.95 versus 20.56 ± 4.21 kg/m^2^, *p* = 0.047), as well as higher estimated glucose disposal rates (eGDR) (9.26 ± 2.01 mg versus 10.55 ± 1.52 mg kg^−1^min^−1^, *p*= 0.0333) [101]. A study on Brazilian patients with DM type 1 (N = 1640) identified MS in 29.8% of individuals, with MS being correlated with AN (OR = 5.93, *p* < 0.001), female sex (OR = 1.95, *p* < 0.001), DM type 1 duration (OR = 1.04, *p* < 0.001), and family history of DM type 2 (OR = 1.36, *p* = 0.019), based on a self-reported color-race model. A second predictive model was used based on self-reported European genomic ancestry and identified a correlation between MS and AN (OR = 6.12, *p* < 0.001) [102]. 

The second youngest child reported (2022) with DM type 2 was a 7-year-old patient from Qatar who was obese and had a family history for DM type 2; this patient displayed severe AN [103]. Previous pediatric programs used AN as a screening tool for DM [104]. There was one study on 151 children (aged between 10 and 14 years) who were screened for DM type 2; the data showed that patients with BMI percentile ≥95% and AN were prone to high blood GGT (γ-glutamyl transpeptidase) as part of IR-related hepatic anomalies [105]. 

A study performed on 320 patients with a mean age of 49.3 years (59.4% females) were found to have normal glucose profiles (N1 = 80) or hyperglycemia (pre-DM or DM representing N2 = 240). AN prevalence was 43.6% (36.3% in N1, 49.6% in N2, *p* = 0.04). The most frequent sites for N1 were knuckles (21.2%) and neck (17.5%), and for N2, neck (29.6%) and knuckles (26.7%). AN specificity predicted IR of 0.85 and 0.9, respectively, and a positive predictive value of 0.86 and 0.96, respectively [106]. The authors suggested that AN sites may be correlated with glucose profile anomalies. 

Trihan JE. et al. found a prevalence of AN of 2.3%, which corresponded to the estimated prevalence for AN among Caucasian populations. Moreover, according to the authors, in diabetic patients, AN could be an indicator of macrovascular complications, such as coronary or carotid artery disease (OR= 2.57, *p* < 0.05) [107]. 

A prospective study on 600 patients (450 obese and 150 healthy controls) found that prevalence of AN was higher in the obese group compared with the control group (47.3 and 3.3%, respectively), with AN being the third most frequent skin finding after striae (64.7%) and acrochordon (52.4%). However, there was no significant difference between diabetic obese patients and non-diabetic obese patients (47.8 versus 46.9%, *p* > 0.05), concluding that DM did not have a major implications in addition to obesity, in terms of skin manifestations; this aspect was not confirmed by other studies [108]. 

For interventional approaches, we investigated metformin as a first-line therapy for DM type 2, especially in obese patients, which seems to act as an anti-inflammatory agent for DM-associated skin disorders, including AN. In addition to glucose profile control, the drug inhibits NF-kB, reduces hyperandrogenemia in females (especially in PCOS), and shows potential antioxidant protection [109]. A systematic review of 64 studies showed that, in addition to a good tolerance profile, there was increased utility of metformin regarding dermatological conditions such as acne, hirsutism (especially in PCOS patients), psoriasis, atopic dermatitis, and hidradenitis suppurative [110]. Alternatively, interventional programs in daily activities proved to be efficient in reducing pediatric obesity and AN, as shown by a large randomized trial on Pacific Islanders [111,112,113]. (Table 2).

## 5. AN and PCOS 

**Figure 1:**
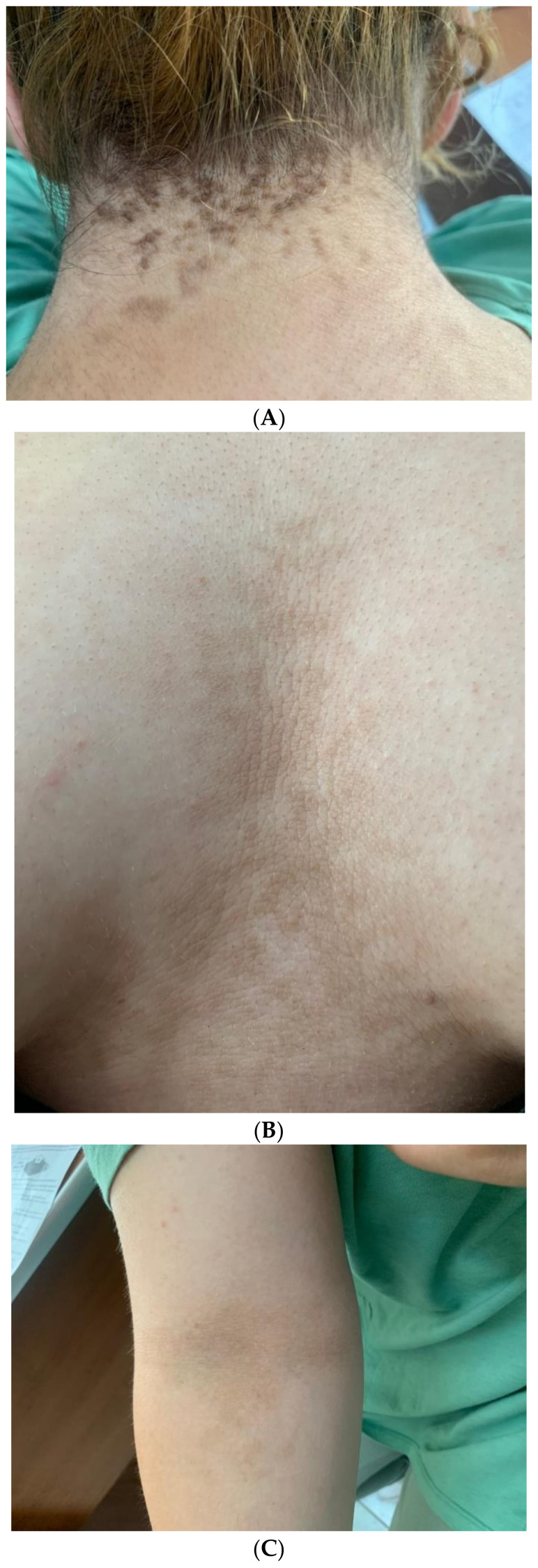

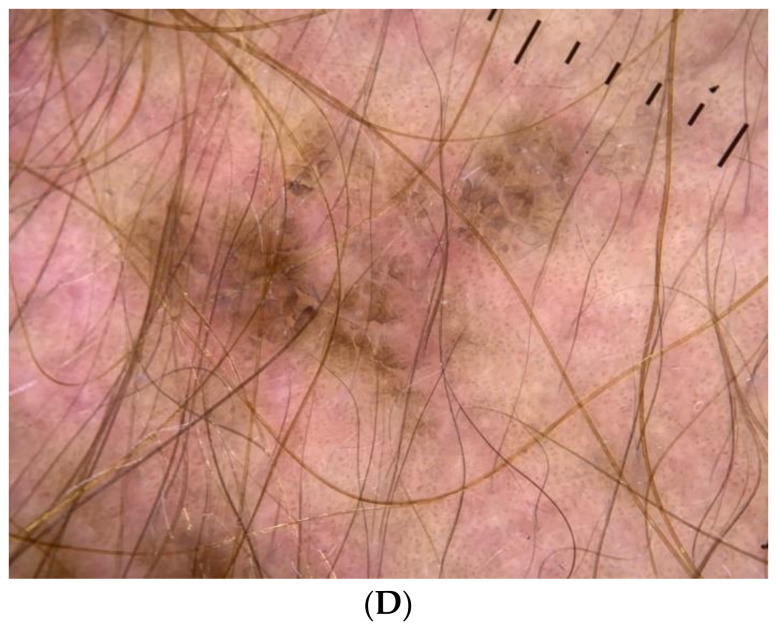
(**A**) A 19-year-old female with obesity and PCOS who presented with neck AN, (**B**) Intermammary and inframammary AN, (**C**) Antecubital fossa hyperpigmented AN lesions; (**D**) Dermoscopy image of AN lesions in the same patient—hyperpigmented dots accompanied by crista cutis and sulci cutis on a papillomatous surface.

The complex clinical spectrum of PCOS may include AN, noting that IR is a common mechanism for both cardio-metabolic features and hormonal dysfunction in PCOS. Females with PCOS may already display metabolic complications, independently of AN. Notably, IR was found even in normal-weighted patients with PCOS (about 20–30%) [114,115]. AN, as well as acne, hirsutism, and high BMI represent the most practical tools to increased awareness of PCOS in addition to menstrual cycle anomalies [116,117]. (Figure 1).

A study on metabolic features in Canadian women diagnosed with PCOS (N = 237 versus 42 controls, aged between 18 and 36 years) showed that MS prevalence was 29.5% in the PCOS group, meaning there was a six times greater risk compared with the control group (*p* < 0.001). The PCOS group experienced more glucose and lipid profile anomalies as well as AN; thus, AN should be considered as part of the clinical signature of PCOS [118]. 

The spectrum of PCOS elements vary. A study on 175 females with PCOS (mean age of 16.8 ± 1.7 years) identified that 77.7% of them had AN; other anomalies included menstrual cycle disturbances (88%), overweight and obesity (69%); high blood pressure (3.4%), different anomalies of glucose profile (24%), hirsutism (94%), with a median Ferriman–Gallway score of 12, and criteria of MS (42%) [119]. 

Another example is a cross-sectional Sudanese study on 368 infertile females with PCOS, which manifested with AN (22%), acne (46%), hirsutism (27%), as well as oligo/anovulation (87%) [120]. A retrospective study on dermatologic findings among 92 patients with PCOS (aged between 11 and 24 years, mean age of 15.9 years) identified 85% of them with AN, as well as acne (93%), hirsutism (38%), alopecia (2%), and hidradenitis suppurativa (16%) [121]. On the contrary, a Chinese study on 186 females with PCOS versus 113 controls without PCOS found acne and hirsutism to be the most frequent findings and AN was the least frequent skin anomaly [122]. Another observational study on 102 subjects with PCOS (aged between 12 and 45 years; 59% in their thirties; mean age of 26.27 ± 5.05 years) identified AN in 50% of them and the following skin anomalies: acne (74%), striae (49%), hirsutism (40%), and almost a third for each of alopecia, acrochordons, and seborrheic dermatitis [123]. A cohort on obese teenagers showed that the subgroup with PCOS had a higher prevalence of AN versus the PCOS-free group (68.9 versus 28.2%, *p* < 0.001) [124]. We concluded that AN rates among PCOS were extremely variable depending on age, weight, pubertal status, PCOS phenotypes (with associated severe hyperandrogenemia or not), presence of IR, obesity, MS or some of its elements, and the genetic traits of a certain studied population.

There were fewer genetic predictions on clinical phenotypes regarding the presence of AN in certain subgroups of women with PCOS. We identified one study on Kashmiri women with PCOS who were investigated on the INSR His1058 C/T (rs1799817) single nucleotide polymorphism, but no particular correlations concerning AN were identified with respect to different genotypes [125]. 

With a prevalence of around 30–50% up to 70–90% among obese PCOS population, AN was one of the most common clinical findings in PCOS, along with acne, hirsutism, and alopecia, being a renowned marker of IR, hyperinsulinemia, and hyperandrogenism. The most frequent sites of AN are the axilla and neck. Although it is still unknown whether IR is a cause or effect of PCOS, the strong connection between AN and PCOS is based on the mechanisms of IR and hyperinsulinemia, which could also explain elevated levels of free testosterone in the PCOS population. Although it is a well-known fact that AN is associated with obesity and IR, there are limited data regarding normal-weighted people with PCOS [126,127,128,129,130,131,132]. As expected, AN was more frequent among obese women with PCOS versus normal-weighted women with PCOS; however, obesity itself is recognized as an independent risk factor for IR, irrespective of PCOS [126]. 

Generally, the frequency of IR in PCOS patients was evaluated to be 60–70%, depending on the assessment used for IR diagnosis. According to Lewandowski SK. et al., HOMA-IR detects more severe cases of IR compared with IRI (insulin resistance index) (*p* < 0.05) [127]. However, bearing in mind that the hyperinsulinemia euglycemic clamp is known as the gold standard to detect IR, Tosi F. et al. published a study comparing the sensitivity and specificity of the M-clamp and its surrogates (HOMA, QUICKI). By using M-clamp, the percentage of women with flawed insulin action was 74.9%, whereas the percentage detected using other methods such as HOMA, G/I, and QUICKI was 41.1%, 48% and 46.7, respectively (*p* < 0.001) [128]. 

In a cross-sectional study that aimed to assess the prevalence and characteristics of cutaneous findings among 146 women with PCOS and their association with hormonal abnormalities, 46 (31.5%) of them presented with AN. All the AN-positive patients had higher levels of BMI (*p* < 0.001) and were more inclined to have four or more PCOS traits (*p* < 0.001), proving that AN was a weight-dependent cutaneous indicator. Moreover, AN-positive women were significantly linked to higher levels of LH and LH/FSH ratios (*p* = 0.003, respectively *p* = 0.021) [129]. AN appears to be a better discriminator of IR than hirsutism and acne. A large, multi-centric, randomized, controlled study on 1000 patients with PCOS, undergoing ovulation induction, found that AN, as well as BMI, WC, and menstrual cycle anomalies correlated with fasting insulin and HOMA-IR, whereas hirsutism and acne score did not [133]. (Table 3).

In terms of interventional attitude, we identified a randomized, double-blinded study on 66 women with PCOS who received either a fixed exercise schedule with oral placebo or the same exercise schedule with metformin for 6 months. Both groups experienced statistical significant changes from baseline regarding the menstrual cycle and BMI, whereas the second group had higher improvement compared with the first group; however, there was no difference for AN and acne between groups [134]. 

## 6. Discussions

### 6.1. AN: The Tip of the Iceberg 

Skin is a reflection of IR-MS and MS-related parameters, with MS being a condition with a massive and dramatic epidemiological impact in modern society. Individuals with IR report not only AN, but also acne vulgaris, androgenic alopecia, atopic dermatitis, hidradenitis suppurativa, and different skin tags. These are also found in individuals with MS with/without androgen excess in females of reproductive age [135,136,137,138,139,140]. 

AN, a clinical marker of IR, is particularly applicable for screening on a larger scale, such as in obese children and teenagers (especially when looking for DM in pre-pubescent children); populations highly susceptible to DM type 2 and MS; individuals with pre-diabetes status and different types of dyslipidemia; and individuals who are referred to primary health care routine controls or do not have easy access to blood evaluations of IR-related spectrum. The clinical assessment of AN is part of a larger clinical picture that also includes very useful data such as BMI, WC, waist-to-hip ratio, and androgen disposition of fat tissue, which may serve as surrogate markers of glucose and lipid profile anomalies, as well as cardiovascular diseases [138,139,141,142,143]. 

The AN spectrum should be taken into consideration with other specific dermatological findings, such as lichen planus and psoriasis that also underline IR. Females with PCOS have potentially associated hirsutism, acne, alopecia, and even hypertrichosis in severe genetic syndromes with IR [144,145,146]. 

As mentioned, diabetic patients may display a well-known palette of skin complications due to persistent high levels of blood glucose, infections, anomalies of immunity, and chronic inflammation (for instance, necrobiosis lipoidica, diabetic dermopathy, scleroderma diabeticorum, acrochordons, keratosis pilaris, bullosis diabeticorum, and granuloma annulare) [147,148,149,150,151,152]. 

It is difficult to determine whether a particular subgroup of patients with IR-MS is prone to skin anomalies; however, we can emphasize the importance of their early recognition to act against multi-organ complications. The evolution of IR-MS may potentially be reversed under adequate control of metabolic features. Strict dermatological intervention is not a rule, but the tip of the iceberg. Based on individual decision, an extensive dermatological evaluation, including biopsy, is sometimes required for positive diagnosis in atypical presentations and for differential diagnosis [35,46,47].

We need further evidence with respect to longitudinal studies, including data regarding the timeline of AN appearance in relation to the entire cardio-metabolic panel; AN reversibility profile, especially in response to nutritional control; and the issue of clinical utility as an IR marker in children and teenagers who have achieved weight control and become normal-weighted after previously having a high BMI.

### 6.2. Endocrine Approach of AN and Beyond 

Other endocrine considerations involve endocrine tumors and different cancers that manifest with AN, but the current level of statistical evidence remains rather low. AN was reported as a pigmented, pruritic rash (with post-biopsy confirmation) on a 40-year-old man with metastatic insulinoma. The role of extremely high insulin levels (as seen in insulinoma), in addition to IR, is part of AN pathogenic mechanisms, but malignant AN (MAN) may be caused by excessive TGF-α levels (which are sometimes massively released by peptide receptor radionuclide therapy, as seen in this insulinoma case) [153]. 

Similarly, in acromegaly, IR is a key contributing factor to AN, and possibly excessive amounts of growth hormone (GH) and IGF1 as well. Patients present a variety of skin manifestations, including IR-mediated skin features, specific lesions related to secondary DM, and dermatologic hallmarks of syndromic acromegaly as seen in multiple endocrine neoplasia type 1 or McCune-Albright syndrome [154,155]. Although the proliferation of dermal fibroblasts is attributed to the increased levels of GH, IGF-1 is responsible for the proliferation of epidermal keratinocytes [156,157]. However, we did not identify any studies particularly addressing the issue of AN in acromegaly. 

AN has been reported in Cushing’s syndrome, a complex conditions which also brings together IR and hyperandrogenemia in women [158,159]. A 12-year-old girl with newly diagnosed celiac disease and Cushing’s disease was admitted with AN [160]. The level of statistical evidence remains low regarding particular aspects of AN and hypercortisolemia [158,159]. Chronic non-tumor-associated hypercortisolemia in patients with IR-MS may be a silent partner to AN, yet we found no study on cortisol levels among patients with AN and metabolic complications. 

With respect to thyroid hormone status, no correlations were established, apart from overlapping data on IR or PCOS [161]. As an extension of the recommendation to use AN in children to screen for DM, one study from 2022 on 677 subjects younger than 18 years of age found that AN was associated with a 3.6 times higher risk of vitamin D deficiency [162]. The result was expected as hypovitaminosis D has been associated with obesity and MS; but this suggests to screen vitamin D status in children with AN, an aspect which is particularly important in young population concerning musculoskeletal growth [162,163].

Another topic included several cases of pseudoacromegaly-related AN [164,165,166]. There was a case of a 12-year-old female with insulin-mediated pseudoacromegaly showing AN, hirsutism, and acromegaly-like appearance. Severe IR was associated to gene mutations corresponding with FGF21 signal transduction pathways, which may be a potential pathogenic loop for AN as well [164]. Two other subjects with pediatric onset were reported in 2018 [166]. Little is known about this unusual entity, but AN represents one of its most accessible signs [165]. Notably, FGFR2 and FGFR3 mutations have been reported in naevoid AN, an exceptionally rare subtype of AN [167,168]. Also, a few case reports identified a mutation of the FGFR3 gene in familial forms of AN, in association with skeletal disorders [169,170].

Beyond traditional endocrine tumors and neuroendocrine neoplasia, MAN has been reported in gastric and pulmonary adenocarcinomas, ovarian and breast carcinoma, cholangiocarcinoma, and liver malignancy [171,172,173,174,175]. One case of a 30-year-old female was admitted for MAN, which was underlying synchronous gastric, pancreatic, and thyroid cancer with rapid progression that, within months, led to a fatal outcome [176]. MAN has a rapid onset, and is not asymptomatic as traditional AN; it is associated with pruritus and may spread to oral cavities with local effects [177]. Its early recognition may help in the management of underlying neoplasia, which is either a very aggressive malignancy with poor outcome or a slowly progressive condition, and MAN may be presented years before cancer detection [178,179,180]. In these cases, a differential diagnosis based on biopsy is indicated [153]. Usually, AN becomes the only clue for a hidden malignancy, and the oral region is usually involved [181,182]. Although the dermatological lesion itself does not display a malignant profile, it behaves similar to a paraneoplastic syndrome [183,184,185].

### 6.3. Specific Dermatologic Management of AN

Wollina U. et al. introduced the idea that AN is like a “two-sided coin”, being either related to IR-MS or malignancies [183,184,185,186]. A patient with AN may experience this skin lesion while appearing asymptomatic or already under surveillance for IR-related components. In case of a newly detected AN, apart from dermatologic assessment, further metabolic or even oncological assessments are required. AN management depends on the underlying condition, and specific dermatologic therapy is generally not required. As mentioned in cases with obesity and DM/pre-DM, lifestyle intervention concerning BMI control from changes in physical activity and diet to bariatric surgery may help. Metformin is the key player in the field of oral medication against DM type 2, and its indication is extended to PCOS and even to AN itself, outside the specific panel of glucose anomalies [82,85,86,109,110]. In MAN, recognition of AN is extremely important, and the patient will require a multidisciplinary approach. 

In terms of cosmetic intervention, short-term trials with melatonin and urea cream have been reported [83,84]. Treatment with topical retinoids, topical vitamin D analogs, oral retinoids, or alexandrite laser may also contribute to AN improvement [187,188,189,190,191]. The downside of applying tretinoin cream is the risk of relapse after ceasing treatment. As such, there is a recommendation for intermittent retinoid application for maintaining clinical results [187,188,189,190,191]. Another topical treatment option are chemical peels with trichloroacetic acid which leads to epidermal destruction, followed by inflammation of the skin and resulting in re-epithelization and rejuvenation [189]. In a pilot study conducted by Zayed A. et al. on six female patients with AN, all of them showed clinical improvement after one month of weekly chemical peel sessions, regardless of the site of the lesions, with no side effects [189]. As far as oral retinoids are concerned, although they are renowned for their efficiency in improving clinical appearance of AN (as well as acne vulgaris with or without PCOS), they have serious disadvantages, such as the necessity for large doses, long treatment period, potential teratogenic effects, and relapse after treatment cessation [29,192,193,194]. Recently, another cosmetic procedure has paved its way in improving AN lesions, namely, the alexandrite laser. In a study conducted by Ehsani A. et al., when comparing long-pulsed alexandrite laser to topical tretinoin-ammonium lactate, the laser group showed a statistically significant reduction in pigmentation (25.67 ± 11.78 versus 18.33 ± 10.63%, *p* = 0.004) [190]. Fractional CO_2_ laser and retinoic acid peel were found to be effective for neck AN [187]. Alternatively, fractional 1550-nm erbium fiber laser was identified as superior to 0.05% tretinoin cream for neck AN in one randomized trial with fewer side effects in the laser group. [191]. Specific dermatologic approaches should be encouraged after achieving metabolic control, especially in cases with severe skin lesions and depending on patient preference for cosmetic improvement. 

## 7. Conclusions

AN represents an important player and surrogate indicator of the already complicated picture of IR. We identified interesting original data within the last 5 years that cover a large area of interest. Awareness of IR-associated clinical features is essential in order to provide prompt recognition of underlying conditions, from obesity to DM and MS to PCOS; AN represents a useful non-invasive surrogate marker of this spectrum in both children and adults. The neck, axilla, and knuckles have been found as the most frequent sites for AN. The pivotal role of exploring this dermatosis could massively improve endocrine and metabolic assessments. A multidisciplinary perspective is mandatory for AN.

## Figures and Tables

**Table 1:** Studies within the last 5 years concerning AN and IR in terms of obesity and MS (order of display is from 2022 to 2018) (please see references no. [43,52,53,54,55,56,57,58,59,61,62,63,64,65,67,68,69,71,72,73,74,76,80,81]).

First Author/ Year of Publication/ Reference No.	Type of Study	Studied Population	Cardio-Metabolic Parameters Addressing AN Assessment
Philip NE. 2022 [56]	Cross-sectional	N = 60 patients with neck AN N1 = 13 patients without MS (21.7%) (mean age: 27.8 ± 8.2 y) vs. N2 = 47 patients with MS (78.3%) (mean age: 35.8 ± 10.9 y)	BMI: N1 = 26.5 ± 3.8 kg/m^2^ vs.N2 = 29.5 ± 3.5 kg/m^2^ (*p* = 0.01) WC: N1 = 87.6 ± 14.1 cm vs. N2 = 104.5 ± 11.7 cm (*p* = 0.0003) Total cholesterol: N1 = 192.2 ± 32.3 mg/dL vs. N2 = 210.2 ± 37.8 mg/dL FBG: N1 = 89.9 ± 6.8 mg/dL vs. N2 = 102.4 ± 22.01 mg/dL (*p* = 0.005) FI: N1 = 9.08 ± 4.3 mIU/mL vs. N2 = 14.09 ± 8.1 mIU/mL HOMA-IR: N1 = 2 ± 0.8 vs. N2 = 3.5 ± 2.2
Afify AA. 2022 [71]	Case-control	N1 = 25 patients with obesity with AN N2 = 25 patients with obesity without AN N3 = 25 healthy controls	Chemerin levels: N1 > N3, N2 > N3, N1 > N2 (*p* < 0.001) 100% of N1 met MS criteria
Alaqil AI. 2022 [52]	Cross-sectional	N = 1525 children (age: 9–12 y) Female/male ratio = 856/669	Cardiorespiratory fitness, AN, HDL-cholesterol, and triglycerides loaded highest on the first component (loadings between 0.42 and 0.57)
Shah VH. 2022 [76]	Case-control	N1 = 40 patients with facial AN N2 = 40 healthy controls	N1 > N2: higher HOMA-IR, insulin levels, FBG, triglycerides, and total cholesterol (*p* < 0.05)
Daye M. 2021 [80]	Case-control	N1 = 108 patients with lichen planus N2 = 109 controls (without lichen planus)	MS: N1 > N2 (50.9 versus 36.7%, *p* = 0.3) AN incidence: N1 > N2 (*p* = 0.009) MS: lichen with AN vs. lichen without AN (*p* < 0.001)
Yamanaka AB. 2021 [53]	Cross-sectional	N = 4023 children (aged between 2 and 8 years)	90^th^ percentile cut points for boys aged 2 to 5 years (58.15 cm) and 6 to 8 years (71.63 cm) were slightly higher than for girls in both age groups
Daye M. 2020 [57]	Cross-sectional	N = 148 obese patients N1 = 59 patients with AN (mean age: 12.29 ± 2.86 y) N2 = 89 patients without AN (mean age: 11.24 ± 2.82 y)	BMI: N1 = 31.9 ± 7.1 kg/m^2^ vs. N2 = 26.08 ± 4.5 kg/m^2^ MS: N1 = 13 vs. N2 = 34 WC: N1 = 103.8 ± 15.6 cm vs. N2 = 97.02 ± 18.5 cm FBG: N1 = 93.17 ± 10.63 mg/dL vs. N2 = 92.70 ± 10.00 mg/dL Insulin: N1 = 27.06 ± 18.45 U/L vs. N2 = 25.64 ± 14.62 U/L HbA1c: N1 = 5.24 ± 1.10% vs. N2 = 5.18 ± 1.28% HOMA-IR: N1 = 5.09 ± 2.25 vs. N2 = 3.73 ± 1.40 TG: N1 = 41.8 ± 8.41 mg/dL vs. N2 = 55.58 ± 47.53 mg/dL Cholesterol: N1 = 162.46 ± 47.7 mg/dL vs. N2 = 160.88 ± 33.97 mg/dL
Özhan B. 2020 [81]	Cross-sectional	N = 40 obese children	DVISS questionnaire: lower urinary tract dysfunction in 19% of cases. Metabolic lab tests were not correlated with the presence of urinary symptoms, only AN (a risk increase of 1.75-fold, *p* < 0.05)
Karadag AS 2020 [43]	Cross-sectional	N1 = 336 patients with AN N2 = 243 healthy controls (AN-free)	AN–BMI correlation (r = 0.299, *p* < 0.001) AN–DM not correlated (*p* = 0.43) Non-DM individuals: AN–WC correlation (r = 0.131, *p* = 0.24) AN–total cholesterol correlation (r = 0.155, *p* = 0.04)
Lopez-Alvarenga JC. 2020. [72]	Cross-sectional	670 Mexican American children (aged between 6 and 17 years, 49% females)	AN: 33% AN heritability: 0.75 (*p* < 0.0001) AN: positive correlation with BMI, HOMA-IR, and CRP (*p* < 0.05) AN: negative correlation with HDL-cholesterol and physical fitness score
Rodríguez-Gutiérrez R. 2020 [63]	Retrospective	628 Mexican children with obesity	IR: 79.3% MS: 55.4% AN was identified in 94.8% of individuals with IR
Das RR. 2020 [73]	Cross-sectional	N = 545 children N1 = 119 patients with MS ( mean age: 11.27 ± 0.20 y) N2 = 426 patients without MS (mean age: 9.57 ± 0.12 y)	BMI: N1 = 23.54 ± 0.20 kg/m^2^ vs. N2 = 20.56 ± 0.11 kg/m^2^ (*p* < 0.01) WC: N1 = 79.77 ± 8.23 cm vs. N2 = 70.06 ± 7.81 cm (*p* < 0.01) FBG: N1 = 99.34 ± 0.54 mg/dL vs. N2 = 83.51 ± 0.30 mg/dL (*p* < 0.01) TG: N1 = 160.10 ± 1.55 mg/dL vs. N2 = 110.91 ± 1.10 mg/dL (*p* < 0.01) HDL-cholesterol: N1 = 35.80 ± 0.29 mg/dL vs. N2 = 44.11 ± 0.15 mg/dL (*p* < 0.01) LDL-cholesterol: N1 = 97.88 ± 0.81 mg/dL vs. N2 = 80.78 ± 0.46 mg/dL (*p* < 0.01) FI: N1 = 22.06 ± 0.64 mIU/L vs. N2 = 10.47 ± 0.21 mIU/L (*p* < 0.01) HOMA-IR: N1 = 5.47 ± 0.17 vs. N2 = 2.18 ± 0.04 AN + : N1 = 89.9% vs. N2 = 34.03%
Singh SK. 2020 [61]	Cross-sectional	N = 70 patients with AN (mean age: 36.48 ± 10.70 y) N1 = 70 controls (mean age: 34.61 ± 11.53 y)	BMI: N = 27.36 ± 3.79 kg/m^2^ vs. N1 = 25.27 ± 3.55 kg/m^2^ (*p* = 0.001) WC: N = 96.48 ± 7.09 cm vs. N1 = 94.17 ± 6.79 cm HbA1c: N = 5.78 ± 0.71% vs. N1 = 5.55 ± 0.32% FG: N = 97.47 ± 23.76 mg/dL vs. N1 = 87.20 ± 9.41 mg/dL (*p* = 0.001) FI: N = 15.97 ± 10.10 μIU/mL vs. N1 = 10.38 ± 3.18μIU/mL (*p* = 0.013) HOMA-IR: N = 4.32 ± 4.44 vs. N1 = 2.27 ± 0.90 (*p* = 0.012)
Rodríguez-Gutiérrez R. 2019 [62]	Retrospective	N1 = 178 infants with obesity without AN (mean age: 16.4 ± 4.8 months) N2 = 49 infants with obesity and AN (mean age: 16.1 ± 4.6 months) N = 227 infants (mean age: 16.4 ± 4.7 months) (control)	BMI: N = 16.47 ± 2.24 kg/m^2^ vs. N1 = 16.22 ± 1.74 kg/m^2^, respective vs. N2 = 17.38 ± 3.39 kg/m^2^ (*p* < 0.001) Family history of diabetes: N = 57.3% vs. N1 = 59%, respective vs. N2 = 51% FI: N1 = 2.42 ± 1.45 μU/mL vs. N2 = 3.67 ± 2.56 μU/mL (*p*= 0.005) FBG: N1 = 77 ± 9.9 mg/dL vs. N1 = 84.2 ± 12.6 mg/dL (*p* < 0.001) HOMA-IR: N1 = 0.46 ± 0.28 vs. N2 = 0.77 ± 0.54 (*p* = 0.001)
Liimatta J. 2019 [68]	Longitudinal	N1 = 30 patients with premature adrenarche N2 = healthy controls	Similar MS prevalence N1 vs. N2 IR: N1 > N2 (*p* = 0.014) AN: N1 > N2 (*p* = 0.01)
das Merces MC. 2019 [55]	Multi-centric, population–based, cross-sectional	N = 1125 primary health care nurses	MS prevalence: 24.4% Most frequent element of MS: low HDL-cholesterol MS-AN correlation (PR = 3.23, 95% CI between 2.65 and 3.92)
Videira-Silva A. 2019 [59]	Cross-sectional	N = 139 overweight patients N1 = 67 patients with AN (mean age: 179 ± 19 months) N2 = 72 patients without AN (mean age: 170 ± 22 months)	BMI z-score: N1 = 2.51 ± 1.34 vs. N2 = 2.49 ± 0.87 WC: N1 = 102.4 ± 14.2 cm vs. N2 = 99.4 ± 12.8 cm FBG: N1 = 84.6 ± 8.2 mg/dL vs. N2 = 84.1 ± 6.5 mg/dL FI: N1 = 26.4 ± 16.3 μIU/mL vs. N2 = 19.0 ± 10.3 μIU/mL (*p*= 0.003) HOMA-IR: N1 = 5.59 ± 3.51 vs. N2 = 4.00 ± 2.29 (*p*= 0.003) Cholesterol: N1 = 159.5 ± 27.7 mg/dL vs. N2 = 152.8 ± 28.0 mg/dL TG: N1 = 94.5 ± 55.1 mg/dL vs. N2 = 79.0 ± 39.3 mg/dL Hyperinsulinemia: N1 = 70.2% vs. N2 = 47.2% IR: N1 = 62.7% vs. N2 = 38.9%
Ribeiro FA. 2019 [69]	Longitudinal	N = 34 females with premature adrenarche	At the age of final height: obesity (11.8%) High blood pressure (8.8%) Hyperinsulinemia (29.4%) Abnormal HOMA-IR (38.2%) AN (14.7%)
Nithun TM. 2019 [74]	Cross-sectional	N = 60 with AN (aged between 2 and 24 y)	AN correlates with IR in normal weighted patients (*p* = 0.045)
Mosimah CI. 2019 [54]	Cross-sectional	CARDIAC project: examination of 52,545 5th grade students in West Virginia to detect AN	AN prevalence: 4.5% of cases 79% of subjects with AN
Palhares HMDC. 2018 [64]	Cross-sectional	N1 = 83 patients with AN (mean age: 11.7 ± 2.9 y) N2 = 78 patients without AN (mean age: 10.8 ± 3.1 y)	BMI: N1 = 27.4 ± 3.4 kg/m^2^ vs. N2 = 23.4 ± 3.6 kg/m^2^ HDL-cholesterol: N1 = 43.0 ± 10.7 mg/dL vs. N2 = 48.1 ± 10.8 mg/dL LDL-cholesterol: N1 = 102.8 ± 28.4 mg/dL vs. N2 = 99.4 ± 32.5 mg/dL TG: N1 = 86.0 mg/dL (41.0–286.0 mg/dL) vs. N2 = 83.0 mg/dL (31.0–445.0 mg/dL) FBG: N1 = 89.2 ± 10.5 mg/dL vs. N2 = 84.1 ± 12.5 mg/dL FI: N1 = 15.1 μUI/mL (4.6–117.2 μUI/mL) vs. N2 = 11.3 μUI/mL (1.4–35.7 μUI/mL) (*p* < 0.0001) HOMA-IR: N1 = 3.2 (0.8–28.7) vs. N2 = 2.3 (0.3–7.1) (*p* < 0.0001)
González-Saldivar G. 2018 [58]	Cross-sectional	N = 294 patients (mean age: 20.2 ± 1.4 y) N1 = 145 patients without AN (mean age: 20.2 ± 1.4 y) N2 = 149 patients with AN (mean age: 20.3 ± 1.4 y)	BMI: N = 24.7 ± 4.7 kg/m^2^ vs. N1 = 23.4 ± 3.7 kg/m^2^ vs. N2 = 26.1 ± 5.2 kg/m^2^ (*p* < 0.001) WC: N = 81.7 ± 13.3 cm vs. N1 = 77.4 ± 11.6 cm, vs. N2 = 85.9 ± 13.6 cm (*p* < 0.001) FI: N1 = 8.6 ± 3.6 μU/mL vs. N2 = 13.5 ± 7.8 μU/mL HOMA-IR: N1 = 1.8 ± 0.8 vs. N2 = 2.9 ± 1.7
Margolis-Gil M. 2018 [65]	Longitudinal	N = 230 obese patients (with a BMI above 95th percentile), aged between 6 and 17.6 y	20.9% met the criteria of MHU MHU patients had a higher IR, as defined by AN + higher HOMA-IR AN at baseline predicted MHU (follow-up for 1 y) (OR = 2.35, *p* = 0.35)
Assunção SNF. 2018 [67]	Cross-sectional	N = 90 patients with AN N1 = 27 patients with obesity and AN(mean age: 11.4 ± 2.3 y) vs. N2 = 63 patients severely obese with AN (mean age: 12.2 ± 2.9 y)	BMI: N1 = 28.25 ± 3.5 kg/m^2^ vs. N2 = 33.57 ± 6.5 kg/m^2^ (*p* < 0.001) WC: N1 = 87.7 ± 9.1 cm vs. N2 = 98.3 ± 13.8 cm (*p* < 0.001) HbA1c high risk for DM: N1 = 33.3% vs. N2 = 66.7%

Abbreviations: N = number of patients; y = years; vs. = versus; AN = acanthosis nigricans; BMI = body mass index; CRP = C reactive protein; DVISS = Dysfunctional Voiding and Incontinence Scoring System; FBG = fasting blood glucose; FI = fasting insulin; HOMA-IR = homeostasis model assessment-insulin resistance; HbA1c = glycated hemoglobin; MS = metabolic syndrome; metabolically healthy obesity = MHU; TG = triglycerides; WC = waist circumference.

**Table 2:** Studies within the last 5 years concerning AN and DM (the order of display is from 2022 to 2017) (please see references no. [93,101,102,105,106,107,108]).

First Author/ Year of Publication/ Reference No.	Type of Study	Studied Population	Cardio-Metabolic Correlations with AN
Calcaterra V. 2021 [101]	Retrospective	N = 138 patients with T1DM N1 = 7 patients with T1DM and AN N2 = 131 patients with T1DM without AN	BMI: N1 = 23.86 ± 4.95 kg/m^2^ vs. N2 = 20.56 ± 4.21 kg/m^2^ (*p* = 0.047) WC: N1 = 72.79 ± 18.92 cm vs. N2 = 69.80 ± 13.53 cm eGDR: N1 = 9.26 ± 2.01 mg kg^−1^ min^−1^ vs. N2 = 10.55 ± 1.52 mg kg^−1^ min^−1^ (*p* = 0.033) Total cholesterol: N1 = 165.64 ± 33.63 mg/dL vs. N2 = 185.00 ± 26.39 mg/dL
Lin V. 2021 [105]	Transversal	N = 151 patients (aged between 10 and 14 y) (T2DM screening)	Patients with ≥ 95% percentile BMI and AN were more likely to have high GGT
Barros BSV. 2021 [102]	Transversal	N = 1640 (Brazilian population with type 1 DM	MS: 29.8% MS-AN: OR = 5.93, *p* < 0.001 (self-reported color-race model) OR = 6.12, *p* < 0.001 (self-reported European genomic ancestry model)
Álvarez-Villalobos NA. 202 [106]	Transversal	N = 320 patients (mean age of 49.3 y; 59.4% women) N1 = 80 with normal glucose profile N2 = 240 with hyperglycemia (pre-DM or DM)	AN: 43.6% (36.3% in N1, 49.6% in N2, *p* = 0.04) AN specificity to predict IR of 0.85 and 0.9, respectively AN positive predictive value of 0.86 and 0.96, respectively
Trihan JE. 2020 [107]	Transversal	N = 213 *p* DM+ (mean age: 67.3 ± 11.9y) N1 = 77 patients with cutaneous signs (mean age: 66.2 ± 12.64y) N2 = 136 patients without cutaneous signs (mean age: 67.9 ± 11.35y)	AN+: N = 5 patients HbA1c: N = 7.16 ± 0.96% vs. N1 = 7.34 ± 0.93% vs. N2 = 7.05 ± 0.95% (*p* = 0.03) Macrovascular disease AN+: N = 5 patients (2.3%) (*p* = 0.024)
Lascar N. 2019 [93]	Retrospective	N = 95 patients with T2DM	AN+: 11 patients (12.4%) BMI: 35.05 ± 9.54 kg/m^2^ WC: 112.5 ± 19.3 cm DM duration: 7.7 ± 3.8 y
Ozlu E. 2018 [108]	Prospective	N = 600 patients N1 = 450 DM+ (mean age: 37.25 ± 11.37y) N2 = 150 controls (mean age: 35.67 ± 11.24y)	AN+: N1 = 213 (47.3%) vs. N2 = 5 (3.3%) (*p* < 0.001) BMI: N1 = 37.22 ± 6.07 kg/m^2^ vs. N2 = 22.23 ± 2.19 kg/m^2^ (*p* < 0.001) WC: N1 = 119.72 ± 12.98 cm vs. N2 = 82.37 ± 9.21 cm (*p* < 0.001)

Abbreviations: N = number of patients; y = years; vs. = versus; AN = acanthosis nigricans; BMI = body mass index; DM = diabetes mellitus; eGDR = estimated glucose disposal rate; GGT = γ-glutamyl transpeptidase; MS = metabolic syndrome; WC = waist circumference.

**Table 3:** Studies within the last 5 years concerning AN and PCOS (the order of display is from 2022 to 2017) (please see references no. [118,119,129,132,133]).

First Author/ Year of Publication/ Reference No.	Type of Study	Cardio-Metabolic Parameters	Hormonal Parameters
Abusailik MA. 2021 Cross-sectional study [129]	N = 146 females with PCOS N1 = 46 AN+ (31.5%) (mean age: 27.0 ± 4.3 y) N2 = 100 AN– (mean age: 26.0 ± 4.7 y)	BMI ≥ 25 kg/m^2^: N1 = 46 vs. BMI ≤ 24.9 kg/m^2^: N1 = 0 (*p* < 0.001)	High LH: N1 = 18 (39.1%) vs. N2 = 16 (16%) (*p* = 0.003) (n = 2.4–12.6 IU/L) High FSH: N1 = 1 (2.2%) vs. N2 = 3 (3.0%) (NR = 3.5–12.5 IU/L) High LH/FSH: N1 = 27 (58.7%) vs. N2 = 38 (38.0%) (*p* = 0.021) (NR < 2) High PRL: N1 = 3 (6.5%) vs. N2 = 5 (5.0%) (NR = 8.4–23.3 ng/mL) High TT: N1 = 12 (26.1%) vs. N2 = 16 (16.0%) (NR = 8.4–48.1 ng/dL) High FT: N1 = 3 (6.5%) vs. N2 = 1 (1.0%) (NR = 0.3–2 ρg/mL)
Kamrul Hasan A. 2021 Cross-sectional study [119]	N = 175 women with PCOS (mean age: 16.8 ± 1.7 y) N1 = 136 (77.7%) AN+ vs. N2 = 39 (22.2%) AN–	BMI: N = 26.3 ± 5.6 kg/m2 WC: N = 85.6 ± 12.4 cm FG: N = 4.9 ± 0.8 mmol/L 2h OGTT G: N = 6.5 ± 1.6 mmol/L TG: N = 136.5 ± 47.6 mg/dL TC: N = 164.9 ± 31.4 mg/dL LDL-C: N = 99.4 ± 25.6 mg/dL HDL-C: N = 37.7 ± 7.5 mg/dL	Testosterone: N = 0.91 ng/mL (NR: 0.51–1.60 ng/mL) PRL: N = 14.97 ng/mL (9.63–22.43 ng/mL)
Kazemi M. 2019 Cross-sectional study [118]	N = 279 women N1 = 237 PCOS+ N2 = 42 controls AN+ : N1 = 123 (51.9%) vs. N2 = 3 (7.1%) (*p* < 0.001)	BMI: N1 = 32.2 kg/m^2^ (31.1–33.3 kg/m^2^) vs. N2 = 23.6 kg/m^2^ (22.4–24.8 kg/m^2^) (*p* < 0.001) TG: N1 = 1.3 mmol/L (1.2–1.4 mmol/L) vs. N2 = 0.8 mmol/L (0.7–0.9 mmol/L) (*p* = 0.001) HDL-C: N1 = 1.3 mmol/L (1.3–1.3 mmol/L) vs. N2 = 1.6 mmol/L (1.5–1.7 mmol/L) (*p* < 0.001) FG: N1 = 5.0 mmol/L (4.9–5.1 mmol/L) vs. N2 = 4.8 mmol/L (4.8–4.9 mmol/L) (*p* = 0.05) FI: N1 = 14.3 μIU/mL (12.7–16.0 μIU/mL) vs. N2 = 5.0 μIU/mL (4.2–5.8 μIU/mL) (*p* < 0.001) 2hOGTT I: N1 = 79.6 μIU/mL (70.2–89.0 μIU/mL) vs. N2 = 35.8 μIU/mL (29.8–41.7 μIU/mL) (*p* < 0.001) 2hOGTT G: N1 = 6.2 mmol/L (5.9–6.5 mmol/L) vs. N2 = 4.8 mmol/L (4.5–5.1 mmol/L) (*p* < 0.001) HOMA-IR: N1 = 2.4 (2.1–2.7) vs. N2 = 0.8 (0.7–0.9) (*p* < 0.001)	LH/FSH: N1 = 2.2 (2.0–2.4) vs. N2 = 1.2 (0.9–1.4) (*p* < 0.001) (days 1–5 of MC) TT: N1 = 2.0 nmol/L (1.8–2.1 nmol/L) vs. N2 = 1.6 nmol/L (1.1–2.1 nmol/L) (*p* = 0.04) (days 1–5 of CM) SHBG: N1 = 38.7 nmol/L (35.5–41.9 nmol/L) vs. N2 = 61.3 nmol/L (53.1–69.4 nmol/L) (*p* < 0.001) (days 1–5 of MC)
Zhang D. 2019 Randomized study [133]	N = 1000 women with PCOS undergoing ovulation induction	AN correlates with FI and HOMA-IR	LH/FSH did not correlate with FI or HOMA-IR
Keen MA. 2017 Cross-sectional study [132]	N = 100 women with PCOS (mean age: 25.18 ± 3.61y) AN+ = 30%	BMI: N = 26.95 ± 4.50 kg/m^2^	LH: N = 7.61 ± 5.34 IU/L (NR:1.9–12.5 IU/L) FSH: N = 4.29 ± 1.61 IU/L (NR:2.5–10.2 IU/L) LH/FSH: N = 1.88 ± 1.16 Testosterone: N = 58.59 ± 24.19 ng/dL (NR: 14–76 ng/dL) DHEAS: N = 124.34 ± 39.47 μg/dL (NR: 61.2–493.6 μg/dL) PRL: N = 15.21 ± 7.26 IU/L (NR: 2.8–29.2 IU/L)

Abbreviations: AN = acanthosis nigricans; BMI = body mass index; MC = menstrual cycle; PCOS = polycystic ovary syndrome; DHEA-S = dehydroepiandrosterone sulfate; FG = fasting glucose; FI = fasting insulin; FSH = follicle-stimulating hormone; GTT = glucose tolerance test; HDL-C = high-density lipoprotein cholesterol; HOMA-IR = homeostasis assessment of insulin resistance; IR = insulin resistance; LDL-C = low-density lipoprotein cholesterol; LH = luteininzing hormone; PRL = prolactine; SHBG = sex hormone-binding globuline; TG = triglycerides; TT = total testosterone; NR = normal ranges, N = number of patients; y = years.

## Data Availability

Data sharing is not applicable to this article.

## Abbreviations

AN	acanthosis nigricans
BMI	body mass index
DM	diabetes mellitus
EGFR	epidermal growth factor receptor
eGDR	estimated glucose disposal rate
FGFR	fibroblast growth factor receptor
FAN	facial acanthosis nigricans
GGT	γ-glutamyl transpeptidase
GH	growth hormone
HOMA-IR	homeostasis model assessment-insulin resistance
IGF	insulin-like growth factor
IRI	insulin resistance index
IR	insulin resistance
MS	metabolic syndrome
MC4R	melanocortin-4 receptor
MODY	maturity-onset diabetes of the young
MAN	malignant acanthosis nigricans
PCOS	polycystic ovary syndrome
SCANS	scoring for AN severity
